# Rapid Recovery From Cortical Blindness Caused by an Old Cerebral Infarction

**DOI:** 10.3389/fneur.2020.00069

**Published:** 2020-02-07

**Authors:** Katsuei Shibuki, Ichiro Wakui, Takeo Fujimura, Masaru Tomikawa, Shin Hasegawa

**Affiliations:** ^1^Department of Clinical Laboratory, Kashiwazaki General Hospital and Medical Center, Kashiwazaki, Japan; ^2^Brain Research Institute, Niigata University, Niigata, Japan; ^3^Department of Internal Medicine, Kashiwazaki General Hospital and Medical Center, Kashiwazaki, Japan; ^4^Department of Neurosurgery, Kashiwazaki General Hospital and Medical Center, Kashiwazaki, Japan

**Keywords:** blindsight, visual cortex, superior colliculus, short-term potentiation, long-term potentiation, amygdala, looming stimulus

## Abstract

When the primary visual cortex (V1) is damaged, cortical blindness results. However, visual information obtained from the superior colliculus (SC) or direct thalamic afferents to higher visual cortices produces unconscious visual functions called blindsight. Alarming visual stimuli suggesting the approach of a predator are known to trigger escape behaviors via visual information mediated by the SC and amygdala in small animals, and salient and dynamic visual stimuli also produce some conscious visual experience even in patients with blindsight. Fresh cortical blindness sometimes recovers spontaneously in patients with fresh cerebral damages, and recovery can be accelerated by early rehabilitation. However, the mechanisms underlying recovery are not well-known. We analyzed a patient with cortical blindness caused by an old cerebral infarction. After repeated presentation of alarming visual stimuli, the ability to detect visual stimuli in the impaired visual field showed behavioral short-term improvement (STI) within a few minutes. Repeated behavioral STI induction was followed by behavioral long-term improvement (LTI) lasting more than several days. After behavioral LTI, the patient partially recovered the ability to read letters presented in the impaired visual field. The behavioral STI experiment, which can be performed within 10 min, may serve as a clinical screening test for anticipating recovery from cortical blindness.

## Introduction

Visual information mediated via the primary visual cortex (V1) is required for conscious sight (1, 2). When the V1 is damaged, the visual fields opposite to the damaged V1 are impaired, a condition known as cortical blindness (3–5). However, patients with cortical blindness have unconscious visual functions called blindsight (6, 7), possibly through visual information obtained from the superior colliculus (SC), which in turn projects to the amygdala (8–10) and the higher visual cortex (11–14). Fresh cortical blindness sometimes spontaneously recovers (15), and the recovery is facilitated by early rehabilitation (16, 17). Possibly, transient malfunctions of cortical areas surrounding the infarction may recover spontaneously, or neural plasticity in the remaining cortical areas may partly compensate for the impaired visual functions (18–20). Another mechanism for recovery is that blindsight is converted to conscious sight by neural synchronization between a neuronal group involved in blindsight, and another neuronal group involved in conscious sight (21, 22). If so, recovery from blindsight may be possible even in a patient with an old infarction, as long as some latent neural pathways between neurons involved in blindsight and those involved in conscious sight remain.

We investigated a patient with cortical blindness caused by an old cerebral infarction. Repeated presentation of alarming visual stimuli such looming discs (23, 24) induced behavioral short-term improvement (STI) of visual stimulus detection in the impaired right visual field. After repeated behavioral STI induction, behavioral long-term improvement (LTI) of visual stimulus detection, lasting for more than several days, was observed. Once behavioral LTI was induced, the patient showed partial recovery in the ability to read letters presented in the impaired right visual field. Although cortical blindness in the present case was caused by an old cerebral infarction, we unexpectedly observed rapid recovery process from cortical blindness as behavioral STI and LTI of visual stimulus detection for the first time.

## Methods

### Patient

**Figure 1:**
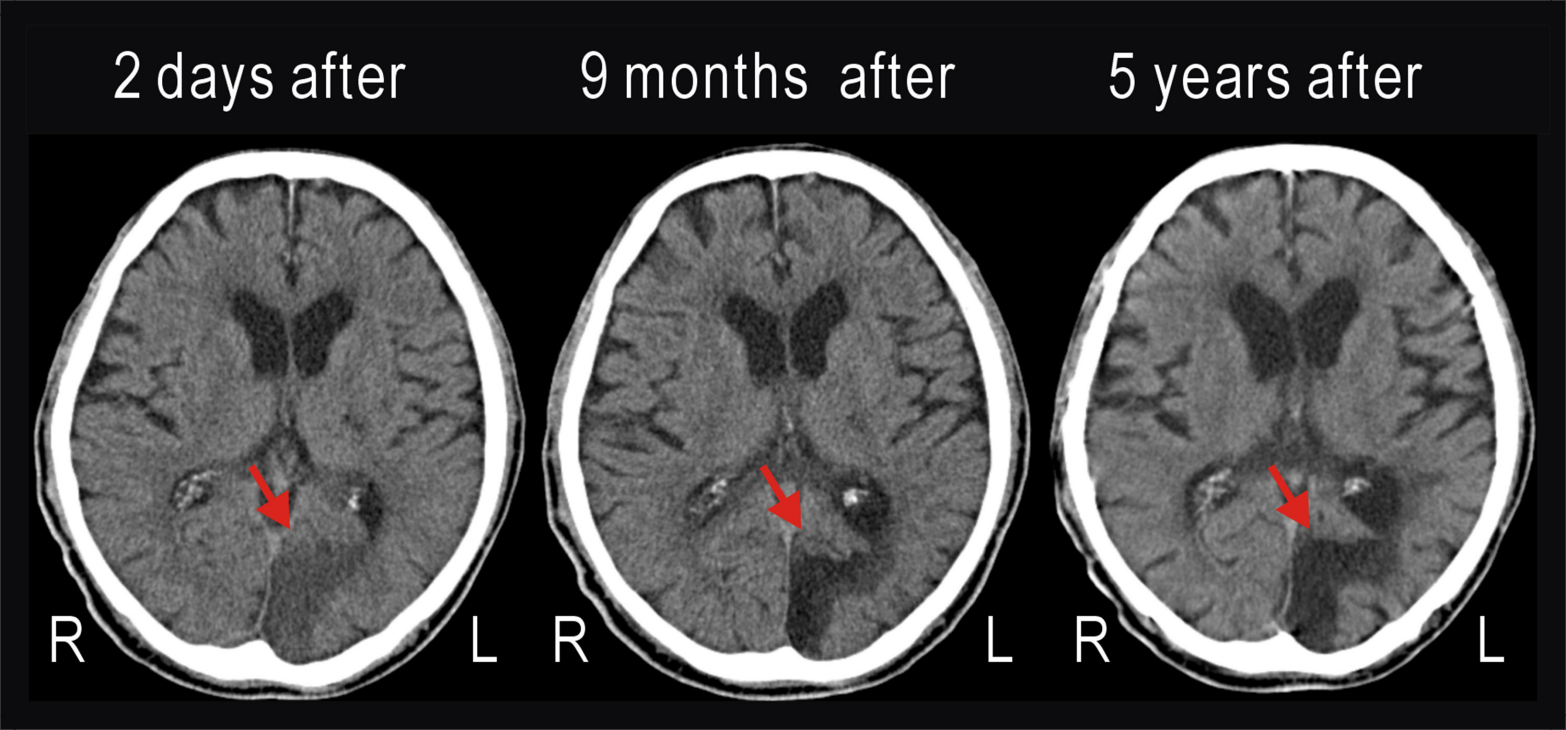
Computed tomography (CT) images taken 2 days, 9 months, and 5 years after cerebral infarction had occurred in the left occipital lobe of the patient. The infarction (red arrow) was unchanged during 5 years.

We investigated an 87-year-old male patient, who suffered from diabetes mellitus and a left occipital lobe infarction occurred 5 years ago (Figure 1). He was admitted to Kashiwazaki General Hospital and Medical Center for glycemic control using hypodermic insulin injection. He was also diagnosed with mild dementia based on Mini Mental State Examination. He could read Japanese Hiragana letters with 24 points (approximately 7 × 7 mm) shown at 600 mm away from him.

### Estimation of Visual Function

**Figure 2:**
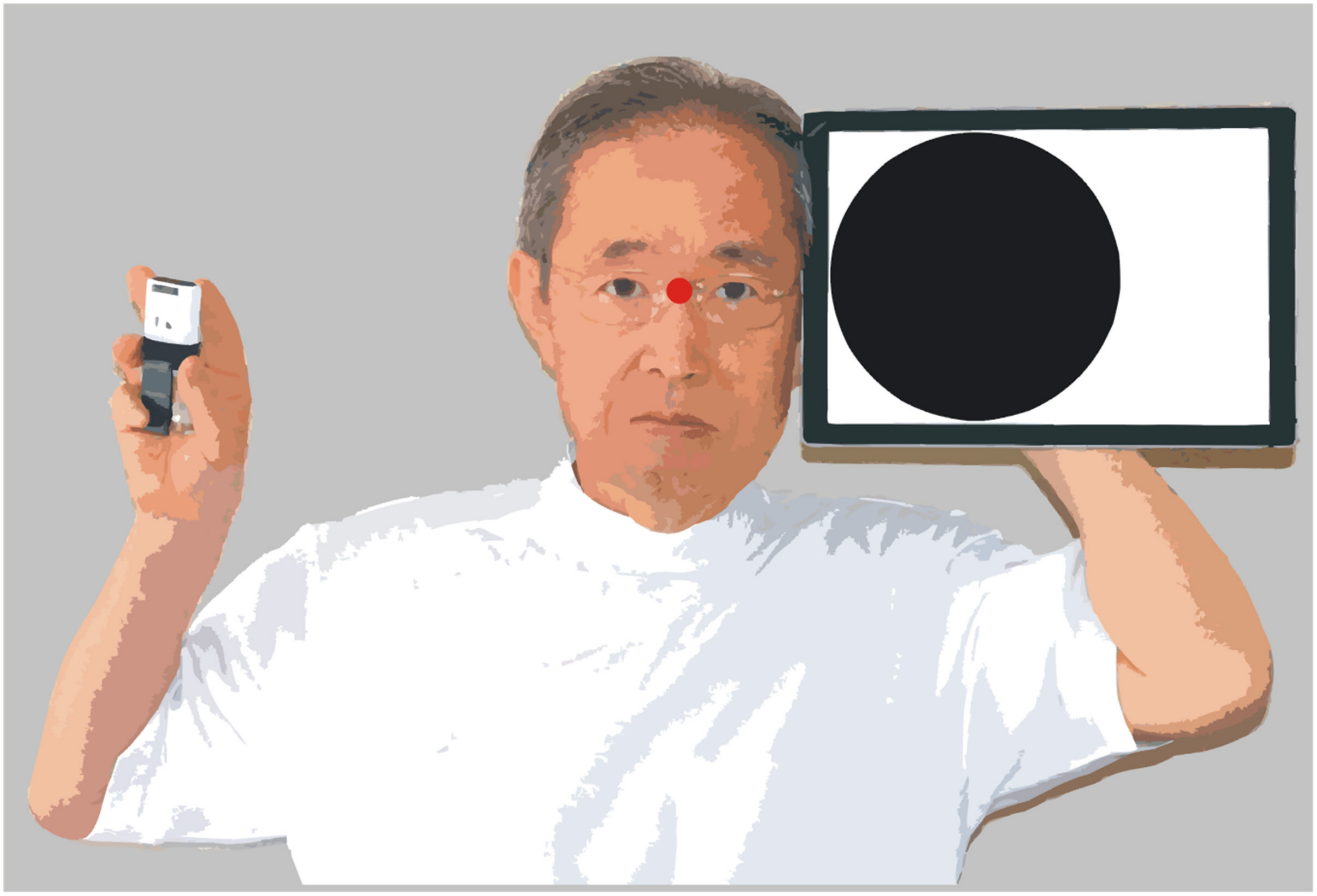
Method for estimating visual functions.

The examiner faced the patient directly at a distance of 600 mm. A tablet computer (Surface Pro 6, Microsoft) was held directly to the left or right of the examiner's face (Figure 2). Various videos created using PowerPoint were presented to the patient. The visual stimuli appeared for 0.5 s in a 188 mm diameter circular range (black circle in Figure 2), the center of which was approximately 200 mm (18.4° of visual angle) away from the fixation point between both eyes of the examiner (red point in Figure 2). When the examiner judged that the patient was looking at the fixation point, the examiner clicked on a small wireless mouse (M-CC2BRSWH, Elecom, Osaka, Japan). The click triggered an animation of the power point file and various visual stimuli appeared for 0.5 s. As soon as visual stimuli were presented, the patient was to report vocally that it was presented, or report a particular property of the stimuli. The mouse was held in a position invisible to the patient, and the operation of the mouse produced almost no sound. Therefore, the examiner could judge that the patient had seen the visual stimuli if the vocal report occurred immediately after clicking the mouse. When examining the control left visual field, the visual stimulus was shown in the symmetrical position.

### Visual Stimuli

The following visual stimuli were used for testing. Static discs: five black disks were shown for 0.5 s (Supplementary Video 1); 400% looming/moving disc: a 47 mm diameter black disc with complicated movements, enlarging to 188 mm in diameter for 0.5 s (Supplementary Video 2); 400% looming disc: a 47 mm diameter black disc with no movement, enlarging to 188 mm in diameter for 0.5 s (Supplementary Video 3); 150% looming disc: a 125 mm diameter black disc with no movement, enlarging to 188 mm in diameter for 0.5 s (Supplementary Video 4); slowly appearing disc: a 188 mm diameter disk changing in color from white to black for 0.5 s (Supplementary Video 5); suddenly appearing disc: a 188 mm diameter black disk suddenly appearing, and changing in color from black to white for 0.5 s (Supplementary Video 6); moving gratings: 20 mm wide vertical stripes with 40 mm wide intervals were shown within a 188 mm diameter circular window, and moved to the right or left by 40 mm for 0.5 s (Supplementary Video 7); random letters: one of 46 Japanese Hiragana letters was randomly selected, and shown with a size of 500 points for 0.5 s (Supplementary Video 8).

### Statistical Analyses

Statistical significance was evaluated using Pearson's χ^2^-test, using Easy R, a free software tool for statistical analysis (25). Correction for multiple comparisons was not carried out, as the original *P*-values (1.1 × 10^−5^ ~ 3.6 × 10^−9^) were sufficiently small.

## Results

### Effective Visual Stimuli for the Impaired Right Visual Field

To confirm the extent of cortical blindness, various static visual stimuli (e.g., static discs, Supplementary Video 1) were shown. The patient could not report stimulus presentation in the impaired right visual field, although the same visual stimuli presented in the control left visual field were noticed with no failures. Next, we presented various dynamic and alarming stimuli in the impaired right visual field. Looming stimuli can be alarming for humans, since the stimuli suggest the presence of rapidly moving objects that may collide with the face. We found that the patient could sometimes notice the presentation of a 400% looming/moving disc (Supplementary Video 2). He reported that something like a black shadow appeared in the impaired right visual field, as reported previously (26).

### Quantitative Estimation of Stimulus Detection

**Figure 3:**
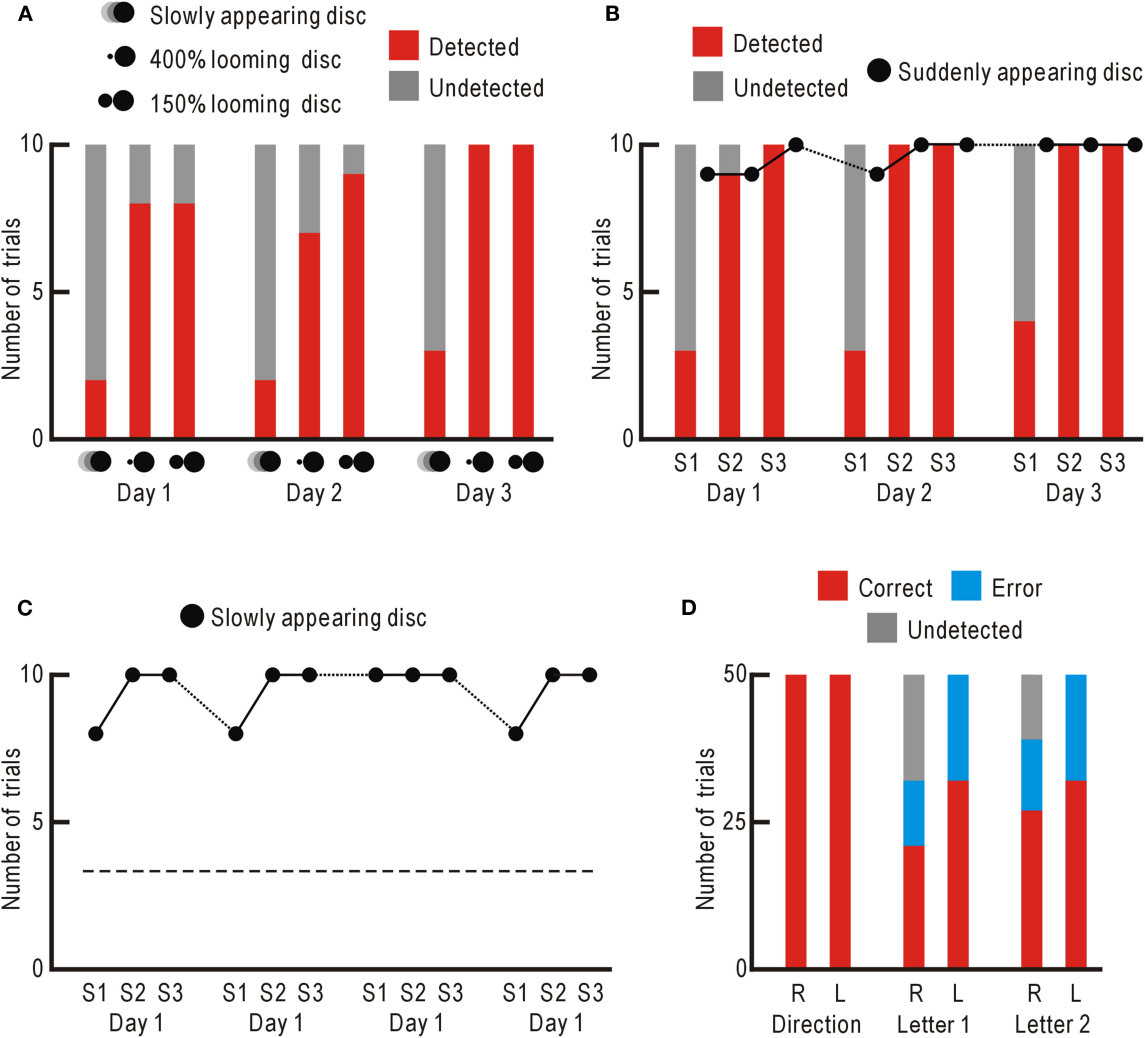
**(A)** Stimulus detection ability in the impaired right visual field. Trials with successful detections are shown in red, and those with failures are shown in gray. **(B)** Behavioral STI of slowly appearing disk detection. For the slowly appearing disc, trials with successful detections are shown in red, and those with failures are shown in gray. For the suddenly appearing disc, only the numbers of trials with successful detections are shown with black dots. **(C)** Behavioral LTI of slowly appearing disk detection. Only the numbers of trials with successful detections are shown with black dots. The averaged detection probability of the slowly appearing disc in **(A)** are shown with broken line. **(D)** Visual perception after behavioral LTI in the impaired right visual field (R) and control left visual field (L). Trials with correct reports are shown in red, those with error reports are in blue, and those with detection failures are in gray.

We found that the patient began to notice the presentation of the static discs in some trials. We used a 400% looming disc (Supplementary Video 3) and a 150% looming disc (Supplementary Video 4), and counted the number of trials with successful detection for 10 trials. As a control stimulus, a slowly appearing disc was used (Supplementary Video 5), since this stimulus had been relatively undetectable. These visual stimuli were presented in the impaired right and control left visual fields 10 times, daily for 3 days (Figure 3A, Supplementary Table 1). The results over the 3 days indicated that the 400% looming disc and 150% looming disc were significantly better noticed compared with the slowly appearing disc (*P* < 1.1 × 10^−5^ and *P* < 7.4×10^−7^, respectively). In the control left visual field, the three stimuli were detected in all 30 trials.

### Behavioral STI of Visual Stimulus Detection

While testing the patient, the slowly appearing disc, which had been hardly noticed originally, began to be noticed more frequently. Therefore, the slowly appearing disc was presented in the impaired right visual field for 10 trials followed by control presentation of a suddenly appearing disc (Supplementary Video 6) for other 10 control trials. This session was repeated 3 times (S1–S3) daily for 3 consecutive days (Figure 3B, Supplementary Table 1). The detection probability of the slowly appearing disc was clearly higher in the second and third sessions, than in the first session. The detection probability over the 3 days was significantly higher in both the second and third sessions, than in the first session (*P* < 1.1 × 10^−6^ and *P* < 2.0 × 10^−7^, respectively). The detection probability of the suddenly appearing disc remained high over the 3 days. These results indicate that the detection probability of the slowly appearing disc showed behavioral STI within a few minutes between the first and second sessions. This behavioral STI had mostly disappeared by the next day. However, the detection probability of the slowly appearing disc in the first session showed a slight increase over the 3 days, suggesting that behavioral LTI of detection probability could be induced under some circumstances. As for the control left visual field, the two visual stimuli were noticed with no failures throughout the 3 days.

### Behavioral LTI of Visual Stimulus Detection

Three days after the behavioral STI experiment, the slowly appearing disc was presented in the impaired right visual field for 10 trials to begin the research on behavioral LTI induction. Unexpectedly, the stimulus was noticed in 8 out of 10 trials in the first session, and in 10 out of 10 trials in the second and third sessions (Figure 3C, Supplementary Table 1). In the first sessions over consecutive 4 days, the slowly appearing disc was noticed in 34 trials and not in 6 trials. This detection probability was significantly higher than that of the results shown in Figure 3A (detected in 7 trials and not in 23 trials, *P* < 5.9 × 10^−8^). These results indicate that behavioral LTI of visual stimulus detection was induced in the impaired right visual field of the patient.

### Visual Perception After Behavioral LTI

We estimated the extent of visual perception after behavioral LTI. We presented moving gratings in the impaired right visual field and randomly moved to the right or left (Supplementary Video 7). The patient correctly reported the direction of movement in all 50 trials (Figure 3D, Supplementary Table 1). The same experiment was performed in the control left visual field, and he again reported the correct direction of movement in all 50 trials.

Next, we presented a randomly selected Japanese Hiragana letter to the impaired right visual field for 0.5 s (Supplementary Video 8), and asked the patient to read the letter. Of the 50 trials, he correctly read in 21 trials, incorrectly in 11 trials, and detection failure was observed in 18 trials (Figure 3D, Supplementary Table 1). As for the trials with successful detection, the correct answer rate (65.6%) was significantly higher than that of random choice (2.2%, *P* < 5.7 × 10^−7^). When the letters were presented in the control left visual field, he correctly read in 32 trials, incorrectly in 18 trials, and no detection failure was observed. The correct answer rate (64.0%) was comparable to the value of 65.6% in the impaired right visual field. The same experiment was repeated the next day. The correct answer rate (69.2%) in the impaired right visual field was significantly higher that of random choice (*P* < 3.6 × 10^−9^), and again comparable to that in the control left visual field (64.0%). This patient could not report presentation of static visual stimuli (e.g., static discs, Supplementary Video 1) in the impaired right visual field before behavioral STI and LTI of visual stimulus detection, and therefore, it is very unlikely that he could read letters presented in the impaired right visual field before behavioral STI and LTI.

## Discussion

Alarming visual stimuli, such as looming or suddenly appearing black shadows, suggest the approach of a potential predator and thus are preferentially detected to trigger escape behavior, even in primitive animals with undeveloped visual cortex (27, 28). In primates including humans, looming stimuli are also strongly recognized (23, 24), possibly because the stimuli suggest the presence of rapidly moving objects that may collide with the face. These stimuli could be perceived via the visual information mediated by the SC and amygdala (8–10, 27) or direct thalamic afferents to higher visual cortices (29, 30), since alarming visual stimuli or salient and dynamic visual stimuli presented in the impaired right visual field were sometimes noticed by the patient, as reported previously (26). The present case is characterized by a series of behavioral STI induction within a few minutes, followed by behavioral LTI persisting for more than a few days, in detection ability of visual stimuli. The time course of functional changes suggests that they are produced by some neural plasticity with similar time course, such as synaptic short-term potentiation (STP) and subsequent synaptic long-term potentiation (LTP) of neural circuits (31, 32). Although many studies have shown positive effects of rehabilitation for cortical blindness (3–5), training is expensive and takes a long time. Furthermore, it may be ineffective for some patients. Therefore, a simple and easy to perform screening test to determine the probability of recovery from cortical blindness is needed. Our behavioral STI experiment, that can be completed within 10 min, may serve as a clinical screening test for anticipating recovery from cortical blindness.

The letter perception experiment shown in Figure 3D strongly suggests that functional recovery is restricted only to the detection ability of visual stimuli in the impaired visual field. Once detected, analysis of visual stimuli was performed with the same accuracy as when the stimulus was presented to the control visual field. These results are well explained by the assumption that neural circuits between neurons involved in blindsight and those involved in conscious sight show repeated synaptic STP followed by synaptic LTP. Synaptic STP and LTP are induced when presynaptic and postsynaptic neurons are activated simultaneously (33, 34). Presentation of alarming visual stimuli activates not only presynaptic neurons involved in blindsight but also postsynaptic neurons involved in conscious sight by changes in arousal level, which are also produced by alarming visual stimuli via the SC and amygdala (8–10, 27). After synaptic LTP has been established, visual information obtained through the SC becomes available for conscious sight neurons, and blindsight can be rapidly converted to conscious sight as a result.

Visual information obtained through the V1 is processed by two different pathways: the dorsal and ventral streams (35). The dorsal stream mainly analyzes movements and spatial information included in visual stimuli (“where” pathway), whereas the ventral stream is important for processing shape and texture of visual stimuli (“what” pathway). The ventral stream extends to the temporal cortex, and contains a group of neurons responding to a specific category of visual objects (36, 37). Visual information mediated via the SC projects to the higher visual cortices of the dorsal stream (11–14). The excellent perception of moving grating direction in the impaired right visual field (Figure 3D) may be explained as a function of this projection. The SC is also known to project to the amygdala, and this pathway seems to process affective shape information such as shadows of predators (11) or expressions on the face (9, 11). However, the perception of Japanese Hiragana letters in the impaired right visual field (Figure 3D) is unlikely attributed to functions of the amygdala. Recently, the presence of other visual pathways from the SC to the postrhinal cortex (38), and from the postrhinal cortex to the ectorhinal cortex and surrounding areas (39) has been identified in rodents. Since the ectorhinal cortex is located ventral to the auditory cortex, it probably corresponds to one of the higher visual cortices of the ventral stream in primates (35). The reasonable perception of Japanese Hiragana letters in the impaired right visual field (Figure 3D) may be explained as a function of human analogs to these murine pathways. The remaining question is which entities of the neural circuits exhibit neural plasticity responsible for the behavioral changes. Possibly, the potentiated synaptic circuits may be dispersed within higher visual cortices that are involved in both blindsight and conscious sight. It has been suggested that visual afferent pathways, which bypass the V1 and directly target higher visual cortices, are strengthened in patients with blindsight (40–43). Activity-dependent changes in these pathways are also likely candidates to explain the present findings. Obviously, however, the present results are far from sufficient to elucidate the underlying mechanisms, and various discussion in the present case report should be tested by further experimental studies on multiple cases of cortical blindness.

## Data Availability Statement

All datasets for this study are included in the article/Supplementary Material.

## Ethics Statement

The protocol of this study was approved by the Local Ethics Committee of Kashiwazaki General Hospital and Medical Center (2019-05-21). This study was carried out in accordance with the recommendations of the Local Ethics Committee and the Declaration of Helsinki, with the written informed consent of the patient for the publication of this case report.

## Author Contributions

KS mainly performed experiments. IW and MT helped with experiments. TF and SH helped with data analyses. KS mainly wrote the manuscript. All authors discussed the results and edited the manuscript.

### Conflict of Interest

The authors declare that the research was conducted in the absence of any commercial or financial relationships that could be construed as a potential conflict of interest.
